# Supplementary material for: Precise timing of transcription by c-di-GMP coordinates cell cycle and morphogenesis in Caulobacter
Source: Nat Commun. 2020 Feb 10;11:816. doi: 10.1038/s41467-020-14585-6 (PMC7010744; doi:10.1038/s41467-020-14585-6)
Supplement: Supplementary file 1 — Supplementary Information [file 41467_2020_14585_MOESM1_ESM.pdf]

SUPPLEMENTARY INFORMATION

Precise timing of transcription by c-di-GMP coordinates cell cycle and morphogenesis in *Caulobacter*

Andreas Kaczmarczyk[1*], Antje M. Hempel[1*], Christoph von Arx[1,3*], Raphael Böhm[2], Badri N. Dubey[2], Jutta Nesper[1], Tilman Schirmer[2], Sebastian Hiller[2] and Urs Jenal[1,4]

[1] Infection Biology, Biozentrum, University of Basel, Basel, Switzerland

[2] Structural Biology, Biozentrum, University of Basel, Basel, Switzerland

[3] Present address: Interregional Blood Transfusion SRC Ltd., Murtenstrasse 133, 3008 Bern

[4] Corresponding author

[*] Authors contributed equally.

Correspondence: urs.jenal@unibas.ch

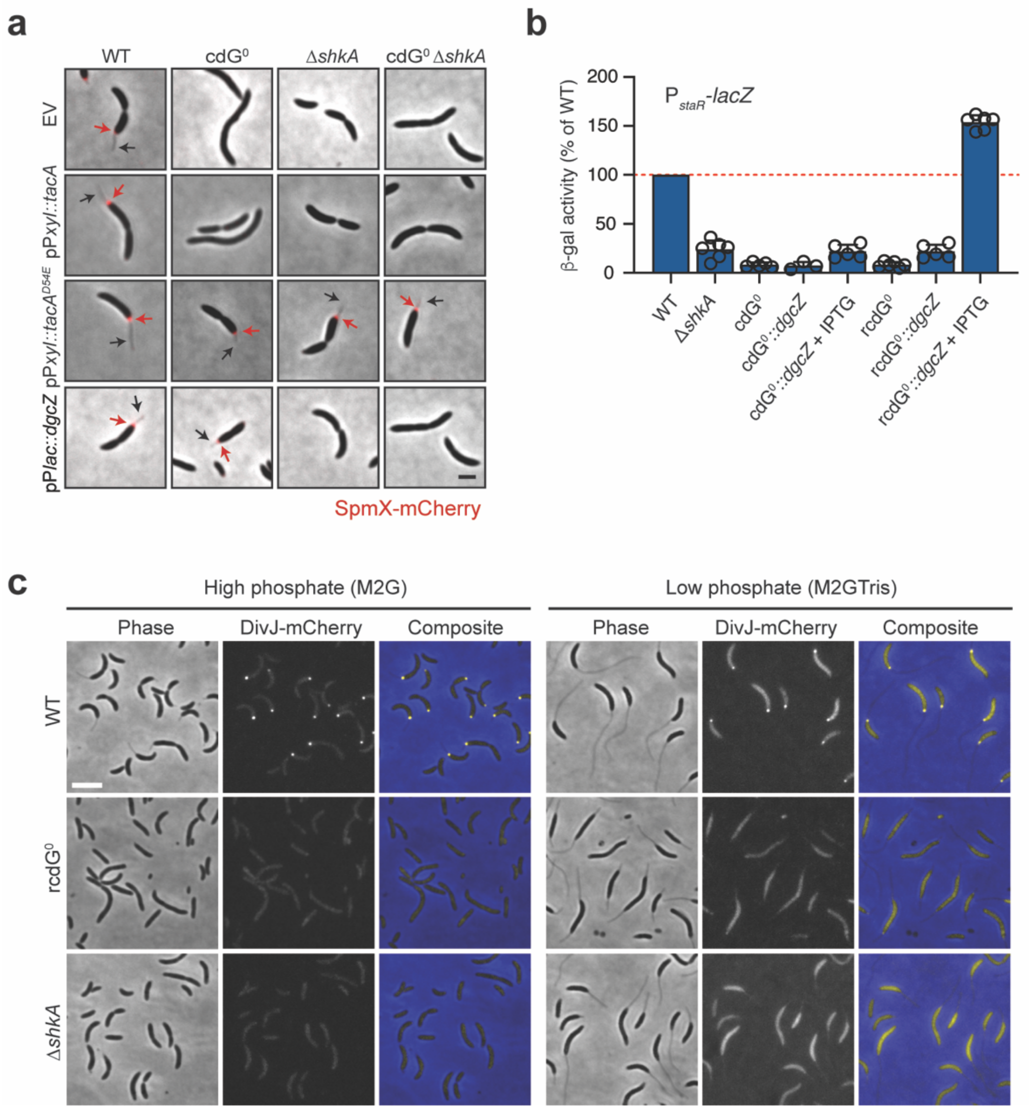

Supplementary Fig 1 – c-di-GMP controls ShkA-TacA activity *in vivo*

a, Micrographs of strains expressing a chromosomal *spmX-mCherry* fusion and plasmid-driven *tacA*, $tacA^{D54E}$ or the heterologous diguanylate cyclase *dgcZ*. EV, empty vector control. Stalks (black arrows) and SpmX-mCherry foci (red arrows) are marked. The scale bar represent 2 µm. **b**, β-Galactosidase activities of indicated strains harboring a P_{spmX}-*lacZ* transcriptional fusion (plasmid pRKlac290-*staR*). "+ IPTG" indicates induction of *dgcZ* expression from P_{lac} by addition of 200 µM IPTG. Shown are means and standard deviations (N>3). **c,** Micrographs of strains expressing a chromosomal *divJ-mCherry* fusion grown under high phosphate conditions (standard M2G medium) or under low phosphate conditions (M2GTris medium, in which the phosphate buffer component was replaced by Tris buffer; see Materials and Methods for details). The scale bar represents 4 µm. Source data are provided as a Source Data file.

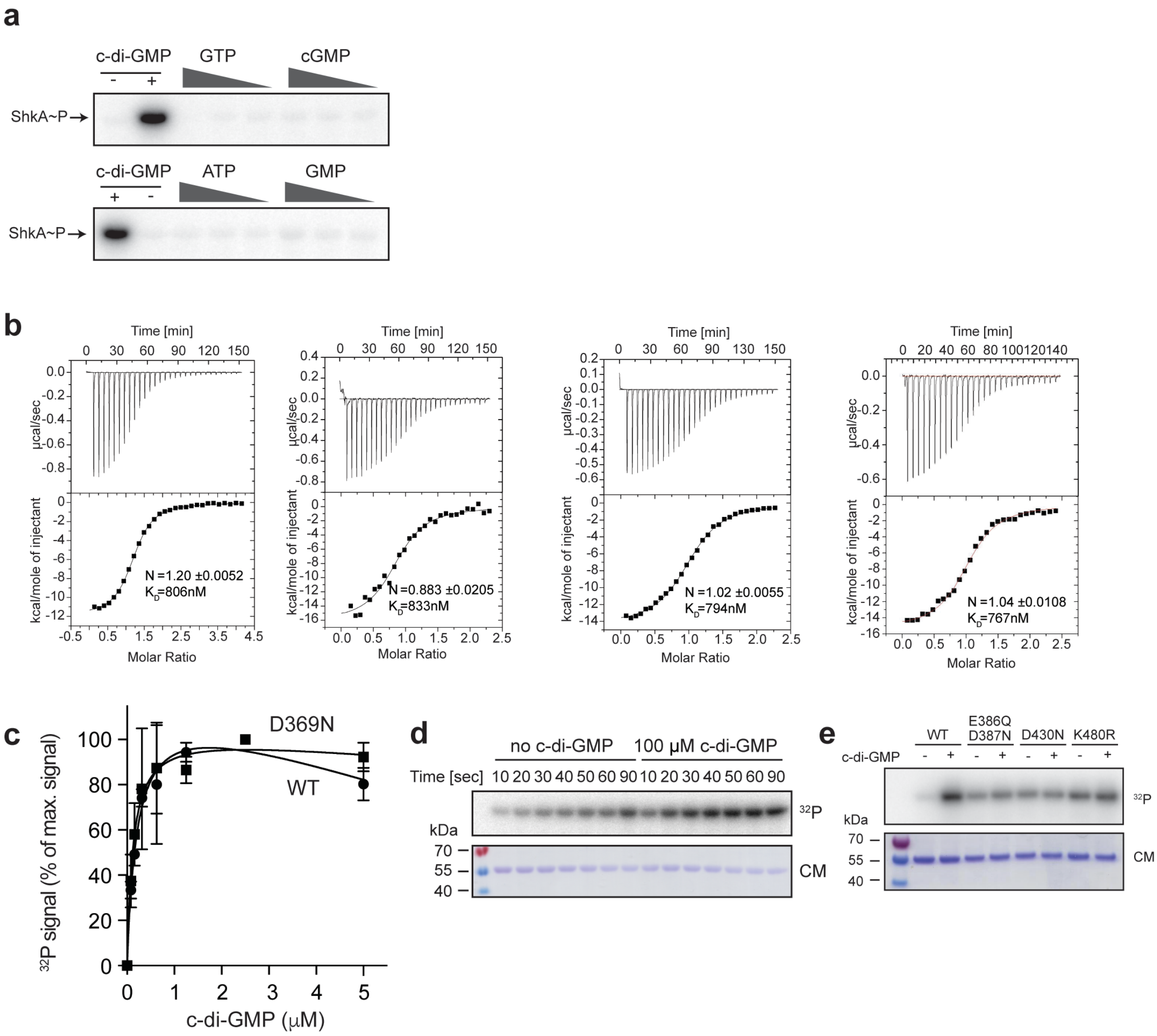

Supplementary Fig 2 – ShkA binds to and activates c-di-GMP *in vitro*

a, *In vitro* phosphorylation assays with purified ShkA in the presence of different concentrations of nucleotides (1 mM, 100 µM, 10 µM). Positive and negative controls contained 100 µM c-di-GMP and ddH_2O, respectively. Reactions were initiated by addition of 500 µM radiolabeled ATP and allowed to proceed for 15 minutes at room temperature. **b**, Isothermal titration calorimetry measurements show a direct interaction of ShkA with c-di-GMP. Four independent experiments are shown. **c**, Quantified autoradiographs of purified ShkA and the ShkA^{D369N} variant (0.5 µM) UV-crosslinked with increasing concentrations of [^{32}P]c-di-GMP. Shown are mean values and standard deviations (N=2). **d**, Time course of ShkA^{D369N} autophosphorylation at 4°C with or without c-di-GMP. Top: autoradiograph; bottom: Coomassie stain of the same gel. **e**, *In vitro* autophosphorylation assays of wild-type ShkA and indicated mutant variants with (10 µM) or without c-di-GMP. Autophosphorylation was allowed to proceed for 5 minutes at room temperature. Top: autoradiograph; bottom: Coomassie stain of the same gel. Residues E386 and D387 are responsible for

magnesium binding of REC2, D430 is the residue accepting the phosphoryl group and K480 plays a role in stabilization of phosphorylated D430. Source data are provided as a Source Data file.

a

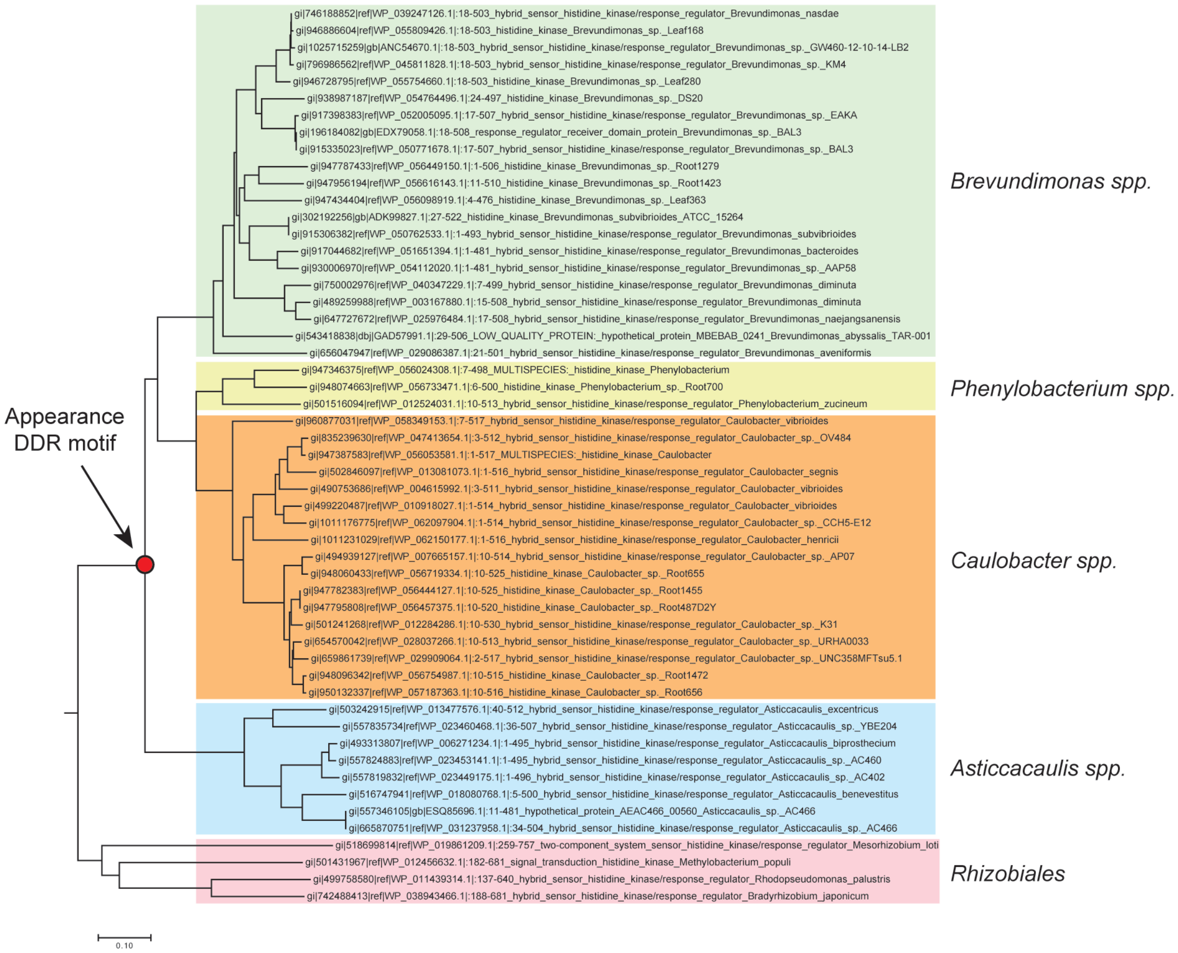

b

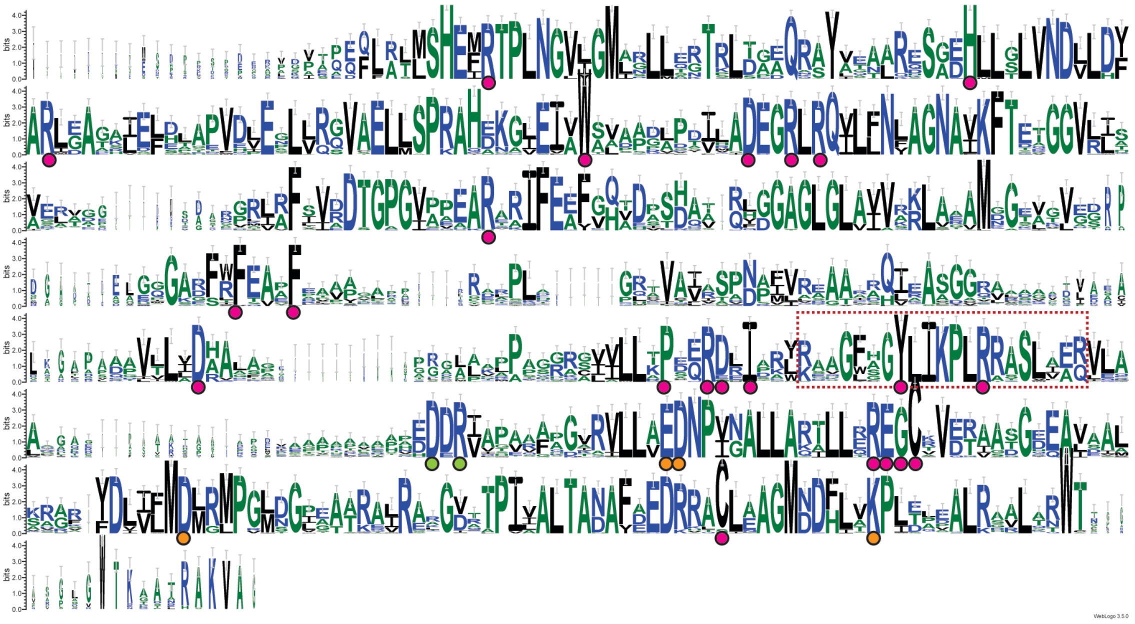

Supplementary Fig 3 – Phylogenetic analysis of ShkA distribution

a, Phylogenetic tree of select ShkA orthologs created with Geneious Tree Builder using default parameters. **b**, Weblogo [1] based on an alignment of ShkA orthologs within the *Caulobacteraceae* shown in **a**. Circles below amino acid residues indicate residues that were mutated and tested for an effect on ShkA activity in *in vivo* (violet), were isolated as mutations that render ShkA activity c-di-GMP-independent (green), or were tested *in vitro* for their role in ShkA autoinhibition (orange). Conserved residues around the c-di-GMP binding site (see Fig. 2j,k) are highlighted by a box.

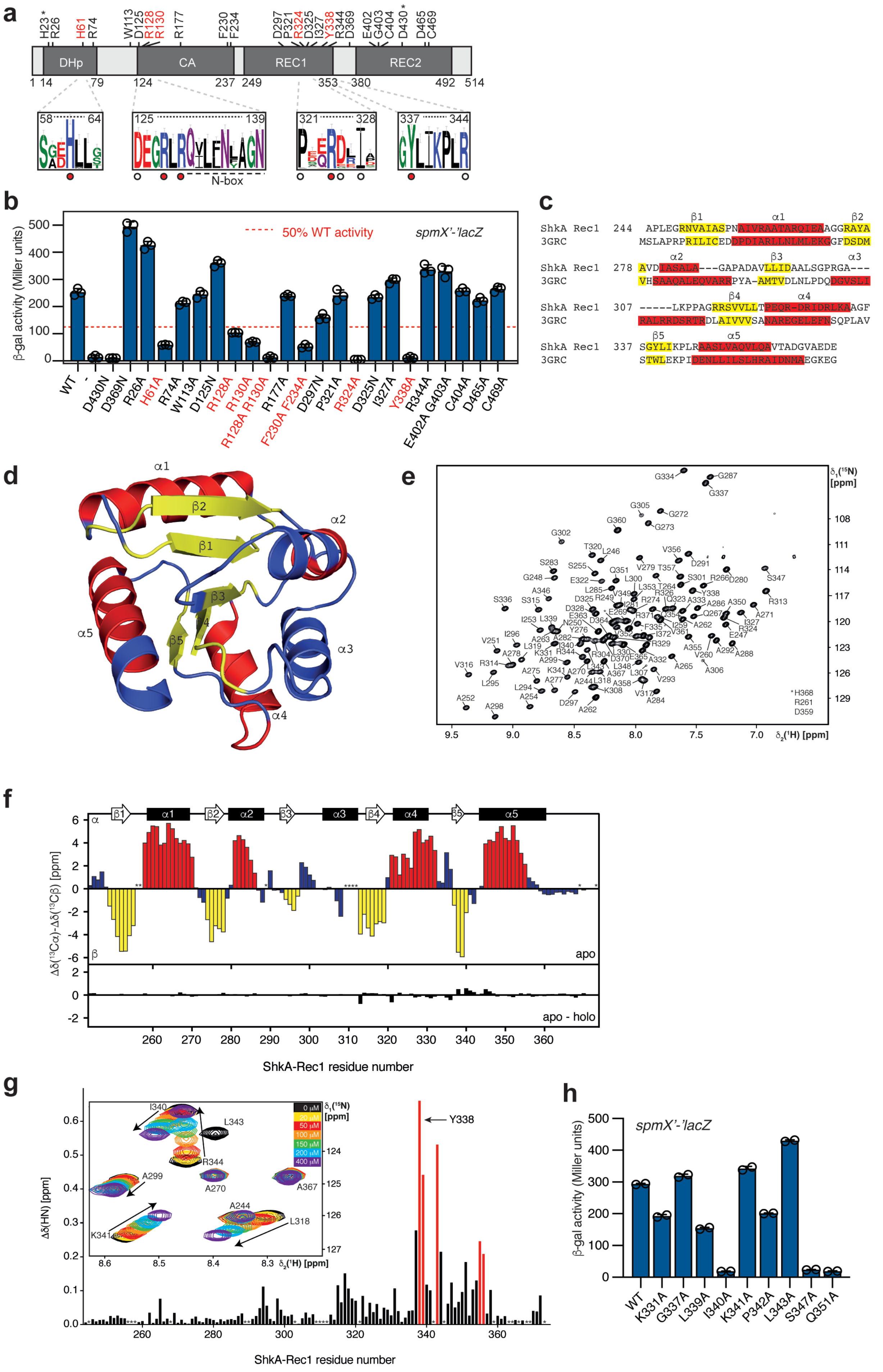
a
H23 * R26 H61 R74 W113 D125 R128 R130 R177 F230 F234 D297 P321 R324 D325 I327 Y338 R344 D369 E402 G403 C404 D430 * D465 C469
DHp
CA
REC1
REC2
1 14 79 124 237 249 353 380 492 514
58 64
125 139
321 328
337 344
N-box
b
β-gal activity (Miller units)
50% WT activity
spmX'-'lacZ
WT
-
D430N
D369N
R26A
H61A
R74A
W113A
D125N
R128A
R130A
R128A R130A
R177A
F230A F234A
D297N
P321A
R324A
D325N
I327A
Y338A
R344A
E402A G403A
C404A
D465A
C469A
c
ShkA Rec1 244 APLEGRNVAIASPNAIVRAATARQIEAAGGRAYA
3GRC MSLAPRPRILICEDDPDIARLLNLMLEKGGFDSDM
ShkA Rec1 278 AVDIASALA---GAPADAVLLIDAALSGPRGA---
3GRC VHSAAQALEQVARRPYA-AMTVDLNLPDQDGVSLI
ShkA Rec1 307 -----LKPPAGRRSVVLLTPEQR-DRIDRLKAAGF
3GRC RALRRDSRTRDLAIVVVSANAREGELEFNSQPLAV
ShkA Rec1 337 SGYLIKPLRAASLVAQVLQAVTADGVAEDE
3GRC STWLEKPIDENLLILSLHRAIDNMAEGKEG
β1 α1 β2 α2 β3 α3 β4 α4 β5 α5
d
α1 α2 α3 α4 α5 β1 β2 β3 β4 β5
e
δ1(15N) [ppm]
δ2(1H) [ppm]
*H368 R261 D359
f
apo
apo - holo
Δδ(13Cα)-Δδ(13Cβ) [ppm]
ShkA-Rec1 residue number
g
Δδ(HN) [ppm]
Y338
I340 L343 R344 A299 A270 A367 K341 A244 L318
0 μM 20 μM 50 μM 100 μM 150 μM 200 μM 400 μM
δ1(15N) [ppm]
δ2(1H) [ppm]
ShkA-Rec1 residue number
h
β-gal activity (Miller units)
spmX'-'lacZ
WT
K331A
G337A
L339A
I340A
K341A
P342A
L343A
S347A
Q351A

Supplementary Fig 4 – NMR analysis of ShkA$_{REC}$

a, ShkA domain architecture (top). Residues that were mutagenized and tested *in vivo* are indicated. Mutants that are impaired in c-di-GMP-dependent ShkA autophosphorylation are highlighted in red. Residues that were mutagenized but did not affect ShkA activity *in vivo* are indicated by a white circle. The phosphor-accepting His and Asp residues of the DHp and REC2 domain, respectively, are indicated by asterisks. **b**, β-Galactosidase activities of strain UJ9691 expressing indicated *shkA* alleles from pQF. Shown are means and standard deviations (N=3). **c**, Profile-profile-based alignment of the REC domains of ShkA$_{REC1}$ PDB 3GRC carried out with HHpred [2]. Secondary structural elements (β-sheet, yellow; helix, red) are indicated as determined by NMR secondary chemical shifts for ShkA$_{REC1}$ in solution (E) and by crystallographic data for PDB 3GRC (C). **d**, The location of secondary structural elements (β-sheet, yellow; helix, red) of ShkA$_{REC1}$ in solution are plotted onto the PDB 3GRC structure. **e**, 2D [^{15}N,^{1}H]-HSQC spectrum of 0.95 mM ShkA$_{REC1}$ recorded at 25°C in 25 mM Tris pH 7.2 with 50 mM KCl and 2 mM $MgSO_4$ in 95%/5% H_2O/D_2O. The sequence-specific resonance assignments are indicated. **f**, Sequence-specific secondary backbone ^{13}C chemical shifts are plotted against the ShkA residue number, smoothed using a 1:3:1 weighting (top). Consecutive stretches with positive and negative values indicate α-helical (red bars) and β-strand (yellow bars) secondary structure, respectively. Asterisks indicate unassigned residues. Secondary chemical shift difference between apo and c-di-GMP-bound ShkA$_{REC1}$ (bottom). **g**, Chemical shift perturbation of ShkA$_{REC1}$ backbone amide moieties upon c-di-GMP binding. Combined chemical shift changes of amide moieties, Δδ(HN), are plotted against the residue number. The red bars indicate resonances that experience intermediate chemical exchange upon c-di-GMP binding. Asterisks indicate unassigned residues. Inset, region of a 2D [^{15}N,^{1}H]-HSQC spectrum from a titration of c-di-GMP to ShkA$_{REC1}$ at 25°C in 25 mM Tris pH 7.2 with 50 mM KCl and 2 mM $MgSO_4$ in 95%/5% H_2O/D_2O. **h**, β-Galactosidase activities of strain UJ9691 expressing indicated *shkA* alleles *in trans* from plasmid pQF. Shown are means and standard deviations (N=2). Source data are provided as a Source Data file.

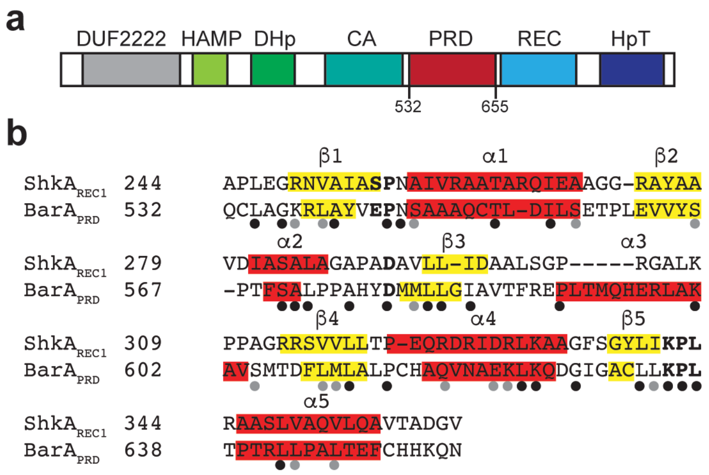

Supplementary Fig 5 – Sequence alignment of ShkA$_{REC}$ and BarA$_{PRD}$

a, Domain architecture of *E. coli* K-12 BarA drawn approximately to scale. Domains were predicted using the SMART web server [3]. The pseudo-receiver domain (PRD) is not predicted by SMART and was manually annotated (residues 532-655) based on the alignment shown in panel b. **b**, Sequence alignment of ShkA$_{REC1}$ and BarA$_{PRD}$ generated with the Geneious alignment tool. Secondary structures (yellow, β-sheets; red, α-helices) for ShkA$_{REC1}$ and BarA$_{PRD}$ are based on NMR data and HHPred predictions, respectively. Residues that are strictly conserved in prototypical REC domains are in bold and identical and similar residues between BarA$_{PRD}$ and ShkA$_{REC}$ are indicated by black and grey dots, respectively, below the sequence alignment. Note that a residue corresponding to the magnesium-binding pocket of canonical REC domains is degenerated in BarA (proline at position 591 instead of an acidic residue found in enzymatically active REC domains).

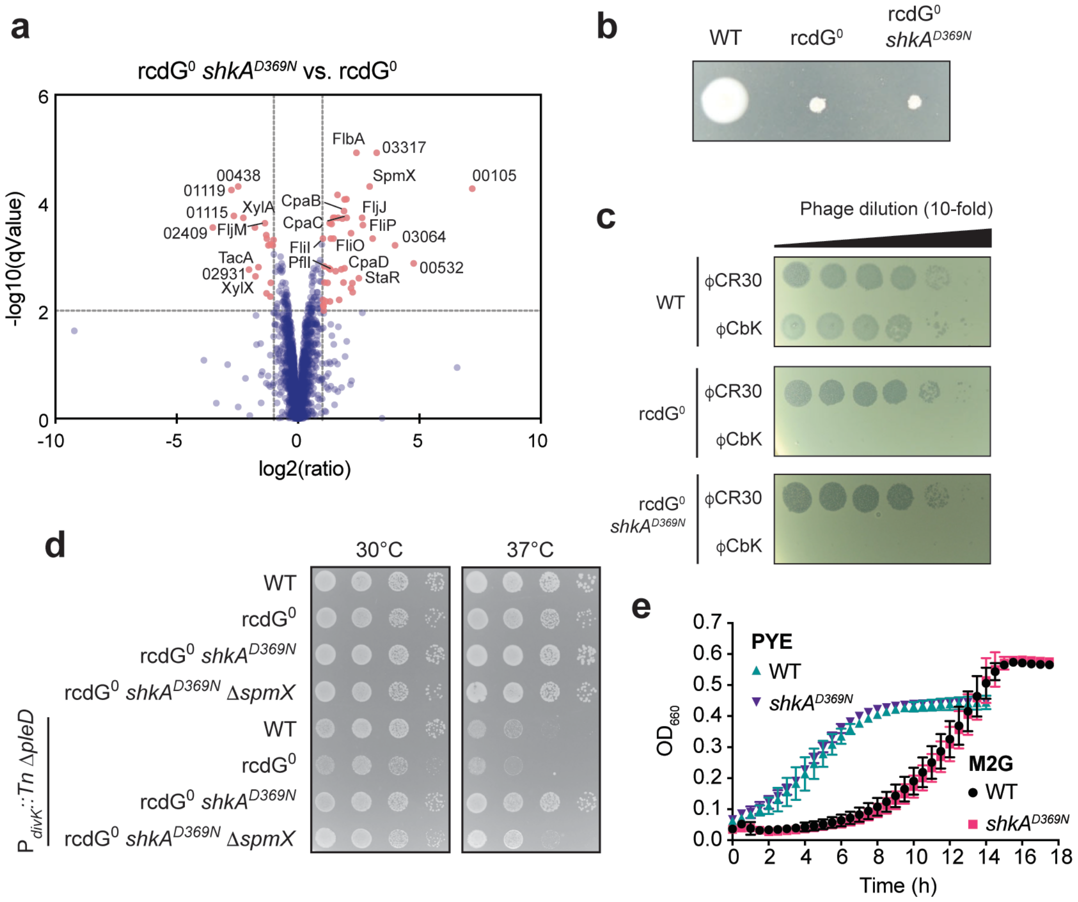

Supplementary Fig 6 – Phenotypic effects of *$shkA^{D369N}$ in vivo*

a, Volcano plot comparing the proteomes of the $rcdG^0$ strain and its derivative carrying the *$shkA^{D369N}$* allele (strains SöA764 and UJ9619). Selected proteins that are differentially abundant in the two strains with high confidence and fold-change are labelled by their NA1000 CCNA number or their annotated gene product. **b**, Motility of indicated strains on semi-solid agar. Plates were incubated 3 days at 30°C. **c**, Sensitivity of indicated strains toward phage ΦCbK or ΦCR30 infection. Plates were incubated 24 hours at 30°C. **d**, Growth of indicated strains on PYE agar at 30°C or 37°C. Strains were 10-fold serially diluted. *PdivK::Tn ΔpleD* was transduced in the different backgrounds from strain SH100 [4]. **e,** Growth curves of wild-type and *$shkA^{D369N}$* strains in PYE and M2G media. Shown are means and standard deviations (N=3).

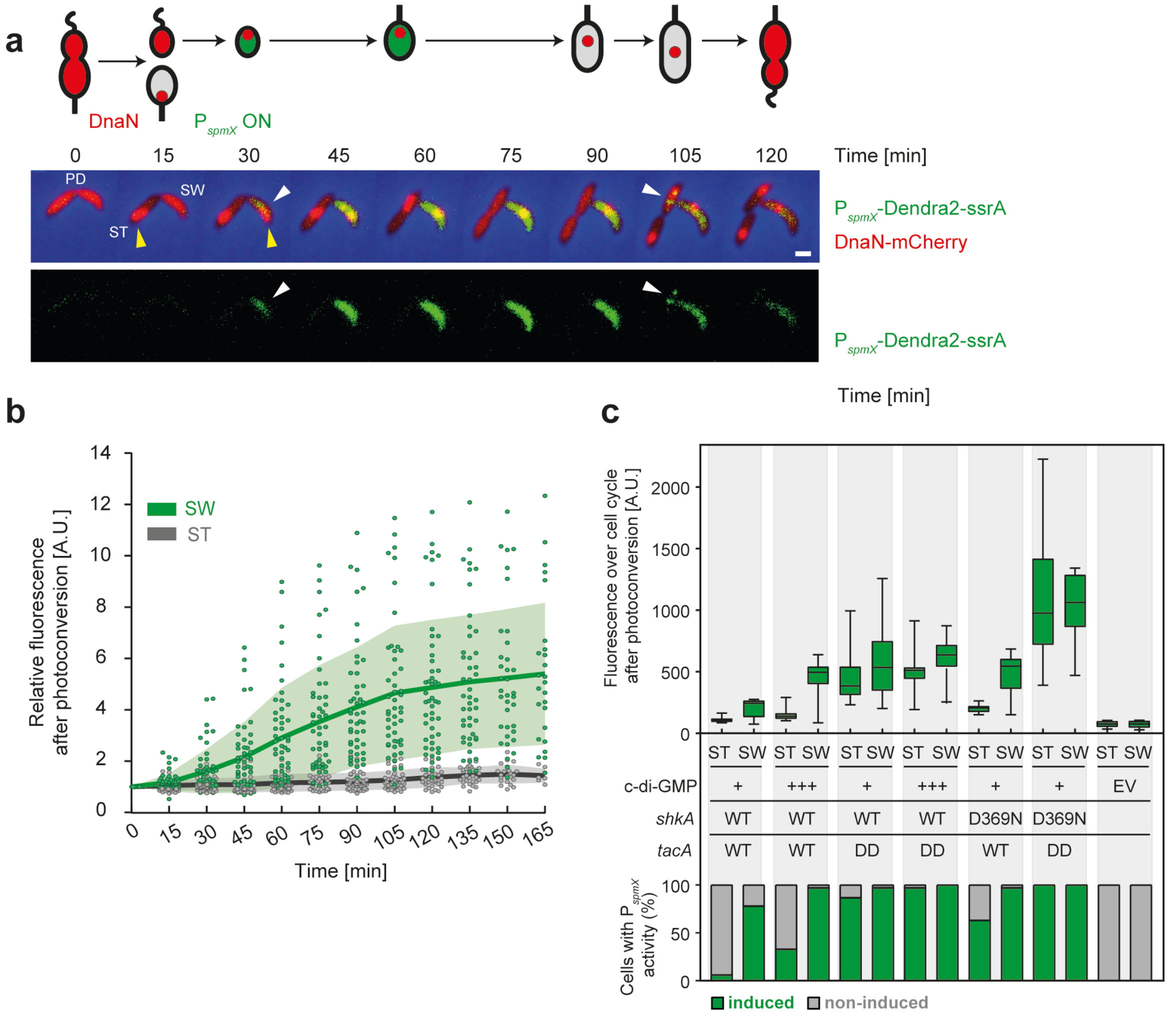

Supplementary Fig 7 – Single-cell analysis of *spmX* expression

a, Representative example of a dividing *Caulobacter* wild-type cell harboring a transcriptional *spmX*::*Dendra2-ssrA* (green) reporter and a chromosomal *dnaN-mCherry* protein fusion (red). Transient localization of DnaN to the replisome at the incipient stalked pole (yellow arrow) indicates the start of S-phase in individual cells. A schematic (top) and fluorescence microscopy images (bottom) of dividing cells are shown at the indicated time points. Note that the *spmX* promoter is activated (Dendra2-ssrA, white arrows) prior to cells entering S-phase. The *ssrA*-tag facilitates proteolytic degradation of the fluorescent protein. The bar is 1 µm. **b**, Fluorescence of newborn wild-type SW (green dots) and ST progeny (grey dots) carrying P*spmX*-*dendra2* after photoconversion in their predivisional ancestors. The fluorescence intensity of individual cells is plotted as arbitrary units (A.U.) (N=50). Mean fluorescence (SW: green line; ST: grey line) and standard deviations (SW: green zone, ST: grey zone) are indicated. **c**, *spmX* promoter activity in newborn SW and ST cells measured by time-lapse microscopy of *C. crescentus* wild type and mutants harboring the *spmX-dendra2* reporter plasmid. For each strain, 30-50 late PD cells were

photoconverted 15 minutes before cell division. Normalised fluorescence of individual SW and ST offspring over the next cell cycle was plotted as arbitrary units (A.U.) in quartiles in box-and-whiskers plots, the median indicated as black line and the whiskers extending from min to max values. The fraction of daughter cells with induced *spmX* promoter activity (green) is plotted in the bar chart below. For experimental details and data analysis see Materials and Methods. C-di-GMP levels were manipulated by P*lac*-driven *dgcZ* with 0.1 mM IPTG. EV, empty vector control.

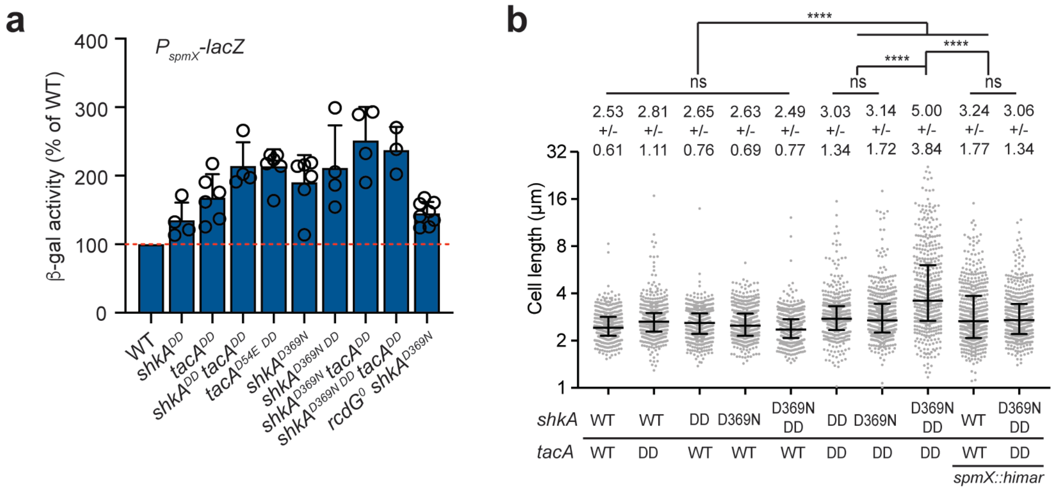

Supplementary Fig 8 – Effects of ShkA and TacA stabilization *in vivo*

a, The expression of *spmX* can be increased by stabilizing proteins in the ShkA-TacA pathway as well as expressing constitutively active ShkA^{D369N}. β-galactosidase assays were performed with strains carrying the pRKlac290-*spmX* reporter plasmid. Promoter activity of *spmX* was measured in wild-type and mutant strains and normalized to wild-type levels. Data represent means and standard deviations (N>3). **b**, Quantification of cell length of strains with mutations in *shkA* or *tacA* that block protein degradation (DD) or with constitutive c-di-GMP-independent ShkA activity (D369N). Median values with interquartile ranges are shown and the means and standard deviations are indicated above the graph. 480-489 cells were analyzed for each strain. *, **, *** and **** indicate P values of < 0.1, <0.01, <0.001 and <0.0001, respectively; ns, not significant (ordinary one-way ANOVA and Tukey's multiple comparison test). Source data are provided as a Source Data file.

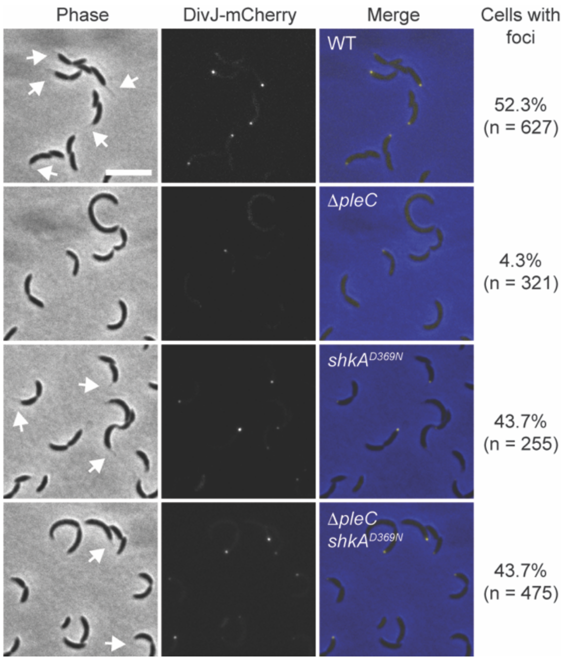

Supplementary Fig 9 – Effect of *shkA*^{D369N} on DivJ localization

Stalk formation and polar localization of a chromosomally encoded DivJ-mCherry fusion was followed in wild-type, *ΔpleC, shkA*^{D369N} and *ΔpleC shkA*^{D369N} strains. White arrows in phase contrast imaged highlight stalks. The scale bar represents 4 μm.

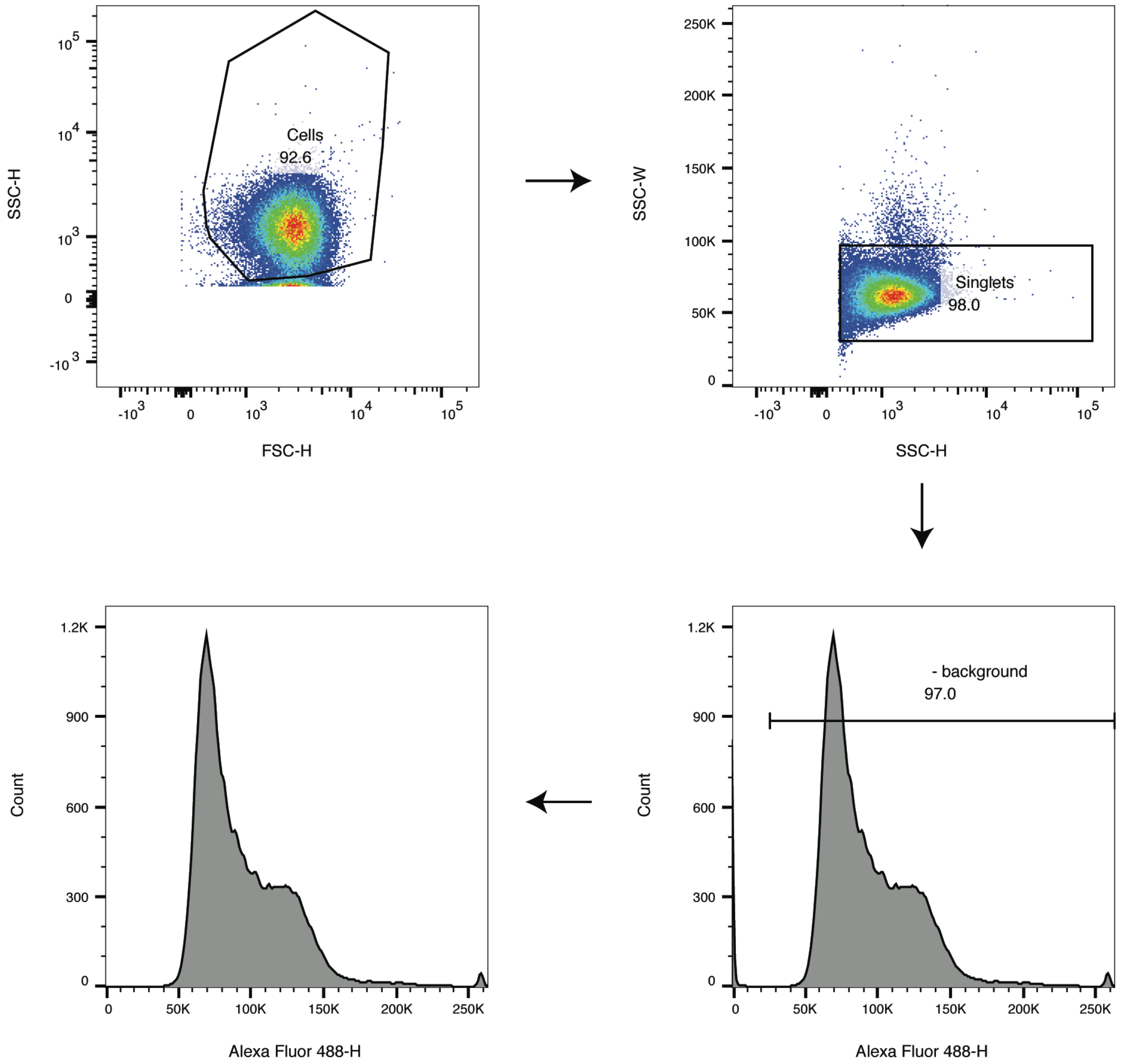

Supplementary Fig 10 – Gating strategy for flow cytometry

Gating strategy for flow cytometry (shown is one of three replicates of the wild type in stationary phase).

REFERENCES

1. Crooks, G. E., Hon, G., Chandonia, J.-M. & Brenner, S. E. WebLogo: a sequence logo generator. Genome Research **14**, 1188–1190 (2004).
2. Hildebrand, A., Remmert, M., Biegert, A. & Söding, J. Fast and accurate automatic structure prediction with HHpred. Proteins **77**, 128–132 (2009).
3. Letunic, I. & Bork, P. 20 years of the SMART protein domain annotation resource. Nucleic Acids Research **46**, D493–D496 (2018).
4. Lori, C. *et al.* Cyclic di-GMP acts as a cell cycle oscillator to drive chromosome replication. *Nature* **523,** 236–239 (2015).
