# Supplementary material for: Precise timing of transcription by c-di-GMP coordinates cell cycle and morphogenesis in Caulobacter
Source: Nat Commun. 2020 Feb 10;11:816. doi: 10.1038/s41467-020-14585-6 (PMC7010744; doi:10.1038/s41467-020-14585-6)
Supplement: Supplementary file 2 — Description of Additional Supplementary Files [file 41467_2020_14585_MOESM2_ESM.pdf]

Description of Additional Supplementary Files

File Name: Supplementary Data 1
Description: **Sequence alignment of ShkA orthologs.** This sequence alignment (in FASTA format) underlies the phylogenetic tree and Weblogos shown in Supplementary Figs 3a, b and 4a.

File Name: Supplementary Data 2
Description: **List of plasmids used in this study.**

File Name: Supplementary Data 3
Description: **List of oligonucleotides used in this study.**

File Name: Supplementary Data 4
Description: **List of strains used in this study.**
